# Functional Inference of Complex Anatomical Tendinous Networks at a Macroscopic Scale via Sparse Experimentation

**DOI:** 10.1371/journal.pcbi.1002751

**Published:** 2012-11-08

**Authors:** Anupam Saxena, Hod Lipson, Francisco J. Valero-Cuevas

**Affiliations:** 1 Faculty Building #369, Mechanical Engineering, Indian Institute of Technology, Kanpur, India; 2 Mechanical and Aerospace Engineering & Computing and Information Science, Cornell University, Ithaca, New York, United States of America; 3 Computing and Information Science, Cornell University, Ithaca, New York, United States of America; 4 Department of Biomedical Engineering & Division of Biokinesiology and Physical Therapy, University of Southern California, Los Angeles, California, United States of America; University of California San Diego, United States of America

## Abstract

In systems and computational biology, much effort is devoted to functional identification of systems and networks at the molecular-or cellular scale. However, similarly important networks exist at anatomical scales such as the tendon network of human fingers: the complex array of collagen fibers that transmits and distributes muscle forces to finger joints. This network is critical to the versatility of the human hand, and its function has been debated since at least the 16^th^ century. Here, we experimentally infer the structure (both topology and parameter values) of this network through sparse interrogation with force inputs. A population of models representing this structure co-evolves in simulation with a population of informative future force inputs via the predator-prey estimation-exploration algorithm. Model fitness depends on their ability to explain experimental data, while the fitness of future force inputs depends on causing maximal functional discrepancy among current models. We validate our approach by inferring two known synthetic Latex networks, and one anatomical tendon network harvested from a cadaver's middle finger. We find that functionally similar but structurally diverse models can exist within a narrow range of the training set and cross-validation errors. For the Latex networks, models with low training set error [<4%] and resembling the known network have the smallest cross-validation errors [∼5%]. The low training set [<4%] and cross validation [<7.2%] errors for models for the cadaveric specimen demonstrate what, to our knowledge, is the first experimental inference of the functional structure of complex anatomical networks. This work expands current bioinformatics inference approaches by demonstrating that sparse, yet informative interrogation of biological specimens holds significant computational advantages in accurate and efficient inference over random testing, or assuming model topology and only inferring parameters values. These findings also hold clues to both our evolutionary history and the development of versatile machines.

## Author Summary

In science and medicine alike, one of the critical steps to understand the working of organisms is to identify how a given individual is similar or different from others. Only then can the specific features of an individual be distinguished from the general properties of that species. However, doing enough input-output experiments on a given organism to obtain a reliable description of its function (i.e., a model) can often harm the organism, or require too much time when testing perishable tissues or human subjects. We have met this challenge by demonstrating that our novel algorithm can accelerate the extraction of accurate functional models in complex tissues by continually tailoring each successive experiment to be more informative. We apply this new method to the problem of describing how the tendons of the fingers interact, which has puzzled scientists and clinicians since the time of Da Vinci. This new computational-experimental method now enables fresh research directions in biological and medical research by allowing the experimental extraction of accurate functional models with minimal damage to the organism. For example, it will allow a better understanding of similarities and differences among related species, and the development of personalized medical treatment.

## Introduction

Much attention is given to functional networks (e.g., scale-free, small world and others) resulting from the complex interactions between their constituents [e.g., 1–5]. For example, the mechanisms of module assembly in biological molecular networks [6]–[8] (with underlying motifs [9]) exhibit coordinated, complex functionalities; interconnectivity among unreliable elements yields reliable dynamic performance [10]–[12]. Similarly, the study of a complex biological system as a whole can be emphasized to understand how system properties emerge from the interaction of multiple components [13]–[15].

Tendon networks at anatomical scales are intricate and poorly understood componentsof the neuromuscular control of the hand. Understanding their functional characteristics is critical to gaining insight into the brain-body co-evolution that has facilitated dexterous manipulation in modern humans, as well as improving clinical rehabilitation strategies in orthopedic and neurological conditions. The complexity of tendon networks of the fingers is legendary, and thus the so-called Winslow's rhombus is a generic topological approximation that has been widely adopted since the 18^th^ Century as proposed by the famous Danish-born anatomist J.B. Winslow in 1732 [16]—especially as the descriptions popularized by Zancolli [17] and Garcia-Elias et al [18]. It is known that minor variations in its structure can exist across humans [e.g., 19], and most work has focused on anatomical/structural descriptions via dissection or imaging and material properties [e.g., 20–27], or simplified computational models [e.g., 28,29]. Importantly, critical structural features, e.g., tendon multiplicity and interconnections are known to remain un-detected with imaging modalities, for example, Ultrasonography (US) or Magnetic Resonance (MR) [30]. As an alternative to structural descriptions, we have proposed functional descriptions of such systems that underscore their sensitivity to topological details [31]. The purpose of this work is to demonstrate that it is possible to use sparse experimentation to, in practice, extract topologies that capture the dominant functional features of these poorly understood structures, which allows us to begin to understand in detail the anatomical and neural co-adaptations that enable dexterous manipulation in modern humans.

This work is enabled and motivated by our earlier work in [32], where we inferred the topology of tendinous networks in simulation. That work showed not only that network topology matters functionally, but that it was—in principle—possible to infer experimentally the structure of arbitrary networks using the most informative force data. Now, we take the critical enabling experimental step of demonstrating the validity and utility of this approach when applied to actual physical anatomical systems, with the imperfections, nonlinearities and actuation/measurement noise that this implies. We do so by testing networks of “known” topology made of strings of synthetic Latex, as well as biological (i.e., cadaveric) tendinous networks of unknown topology. As mentioned above, these biological specimens are complex sheets of collagen fibers, which for centuries have been approximated as networks of strings [16], but for which there is no functionally validated string-based approximation.

## Methods

We describe (i) the development and implementation of the estimation-exploration algorithm for arbitrary networks of strings, and (ii) its application to the experimental inference of two known networks made of synthetic Latex, and one extensor mechanism network excised from the middle finger of a cadaveric human hand.

**Figure 1:**
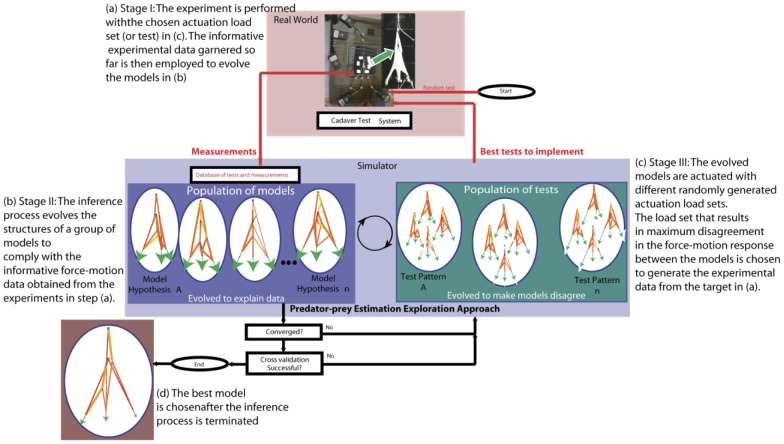
Concept depiction – the structural constitution of the hidden complex tendinous networks (e.g., inset in a) can be inferred via the most informative force-motion data. Steps (a)–(c) are performed cyclically until a termination criterion is met after which the best model (d) is chosen. (c) represents the evolved models from step (b) being actuated by a random set of input forces (or tests) shown using arrows. The length of each arrow represents the magnitude of the force applied. The actuation directions may or may not be held fixed (here, shown fixed). The actuation force set that generates the maximum discrepancy in the force-motion response between the evolved models is chosen to perform the experiment in (a).

The key concept in the estimation-exploration inference process ([33]) is to infer a functional model of the physical network in an informative way that minimizes testing time and potential damageto the tissue—both critical when testing perishable or fragile biological specimens and systems. This three-stage process begins with the collection of a few random input-output force data sets (i.e., **loadsets**) from the network to seed the database of load sets (**Stage I**, Fig. 1a). **Stage II** is a modeling process to synthesize a population of candidate functional models that best replicate the first random and then the informative data acquired in **Stage I**. The fitness of each model is calculated by performing forward simulations to predict how well the model can replicate the available load sets in the database (Fig. 1b). **Stage III** is a complementary modeling process to synthesize a population of candidate force inputs to apply to the experimental system next (i.e., to generate the most informative future load sets, Fig. 1c). The fitness of each input is given by the disagreement (i.e., variance) it produces in output responses of the models synthesized in **Stage II**(i.e., an input that produces similar outputs across all models is the least informative test to perform). In **Stage I**again, the predicted, most-informative input is applied to the network system (Fig. 1a). This adds a new, presumablymost informative, load set to the database whose subsequent impact on the fitness of previously evolved models leads to the synthesis of a new population of models that is on average more compatible with the available experimental data. This inference method is in essence a predator-prey co-evolutionary process between models and the most informative tests that promotes both accurate functional models and efficient experimental testing thatcontinues until the termination criteria are met.

We first describe the modeling processes for Stages II and III, which are identical for the synthetic Latex and cadaveric networks because they are both modeled as networks of elastic strings. Details of how we measureand apply input/output data to generate load sets in Stage I forboth experimental systems are described thereafter. We assume that the mechanical function of the target network system is well approximated by a model of discrete, interconnected/sliding strings. We further assume that all the input and output nodes are accessible for otherwise hidden networks, as is the case for the synthetic and anatomical networks tested. The state of a hidden network is described by a set of actuating forces at the input nodes, those resulting at the grounded output nodes, and the distances between the input and output nodes after the network attains equilibrium. Input and resultant forces comprise the load set while the inter-nodal distances comprise the deformation data. Both force and deformation data are used to evolve the models to best explain a network since ignoring either information may result in more number of experiments (in Stage I), see supporting information S1.

### Stage II: Simultaneous topological and parametric evolution of models in simulation

**Figure 2:**
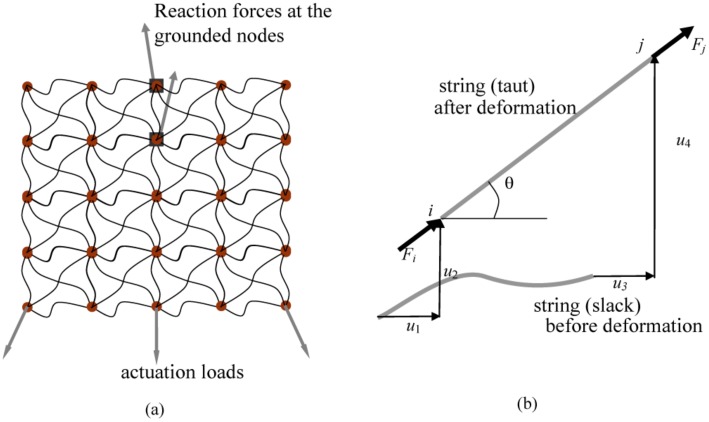
Models are evolved from the primordial mesh of strings in (a). Length of each string behaves as a topological and parametric design variable. For the informative equilibrium configurations that the mesh observes, if some strings remain slack, they are eliminated from the topology. Only taut strings in some or all configurations get retained. Length and cross sections of strings are evolved as design variables. (b) Each taut string in the parent mesh is modeled as a large deformation truss finite element. Slack strings do not support the external loads. Analysis performed is geometrically (for synthetic targets) and materially (for the finger extensor mechanism) nonlinear and equilibrium is achieved through efficient, quadratically convergent Newton-Raphson iterations.

We evolve a population of models (both topology and their parametric attributes) bottom-up from a primordial mesh of strings (Fig. 2a). The strings are arranged such that some are joined at the nodes (shown as filled circles in Fig. 2a), while the rest overlap and slide past each other. This connectivity allows the models to evolve into any topology (i.e., number of strings and intermodal connectivity) that a hidden network system may have. The length and cross sectional area of each string are used as free parameters to evolve the model topology and parameter values simultaneously. String length is a topological as well as a parametric variable: a string can be considered absent from the model if it evolves to a length for which it remains slack for all loading conditions; or present if taut for some or all load setswhere its length influences force transmission and inter-nodal distances. Cross sectional area is a parametric variable that defines the load-bearing and deformation characteristics of a string for given stress-strain relationship (linear or nonlinear). For synthetic networks in this work, we used the linear stress-strain relations for Latex rubber (part SLR-040-E, 1 mm thickness, 380 mm×305 mm, Small Parts Inc.), while for the tendinous extensor mechanism we employed nonlinear tendon properties as reported in [34]. The total number of nodes is maintained constant, but their location is allowed to vary in response to loading—except for the two grounded nodes (shown as filled squares in Fig. 2a) where reaction output forces are measured.

Stage II assumes access to the most informative database of load sets, and consists of four parts, (i) *population of models*, (ii) *model analyses*, (iii) *theirfitness evaluation*, and (iv) *evolution*.

- **Population of models.** We evolve eight models for each synthetic network and five models for the finger extensor mechanism. The models are evolved independent of each other (i.e., no information exchange is permitted among them). While large populations of models are desirable, the computational cost increases with every model introduced. As a proof of concept, we were able to evolve small populations of eight and five models respectively within 24 hours using the available computational resources. As mentioned before, each model stems from a primordial mesh of strings (Fig. 2a). The length and cross-section of each string in a model are optimized (see below) to explain all available load sets in the database. In each model, the three movable input nodes and the two grounded output nodes are pre-specified in the primordial mesh (Fig. 2a). We ensure that the distances between the grounded nodes in the target network and those in the models are identical. Initial distances among the input nodes can however be different from those in the target networks. This is because we assume that all strings are slack before the model is actuated.
- **Model analysis.** To estimate how well a model explains the experimental data, wesimulated the deformation and force transmission for a given set of input forces. Given the material properties of the Latex and tendinous tissue we used, the strings undergo large displacements but small strains. To solve for displacements and force transmission, we used efficient, quadratically convergent, geometrically and/ormaterially nonlinear finite element analysis [35], [36]. Taut strings in a model behave as truss finite elements while slack ones do not contribute to tension propagation and are ignored in the force and stiffness calculations. A model string of resting-length *l*_o_ and cross section area *A* is shown in tension in Fig. 2b. The initial coordinates of the end nodes *i* and *j* are (*x_i_*, *y_i_*) and (*x_j_*, *y_j_*) and the nodal displacements along the horizontal and vertical directions respectively are (*u*_1_, *u*_2_) and (*u*_3_, *u*_4_) as shown. The new string length is
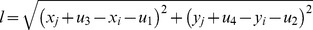
For the Latex networks, the stress 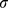, and the strain∈ are related as 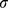 = *E*∈ where *E*, the elastic modulus isassumed constant (1.62 MPa) over a large deformation range. This value is verified experimentally for Latex string members subjected to small strains. The stress vs. strain properties for the human tendinous tissue from [34] are stated below.
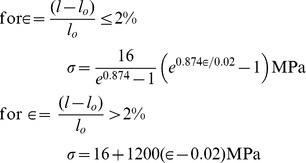
For synthetic Latex, internal axial forces *F_i_* and *F_j_*at the two nodes *i*and *j* are computed as 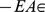
and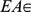. For the tendinous tissue, they are
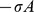
and 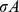. In case *l*<*l*_o_, since the string collapses in compression, both *F_i_* and *F_j_* are zero. For a taut string, the force (**f**) and displacement (**u**) vectors are
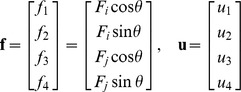
The local internal forces in **f** are assembled within **F**, the global internal force vector as in the conventional finite element assembly [35]–[36]. Input forces (i.e., the most informative tests) actuating the network are recorded through the external force vector **F** *_e_*. We employ Newton-Raphson iterations to solve for the equilibrium equations. The force residual, **g**(**U**) = **F**−**F** *_e_*, is expanded through its first order Taylor's approximation.
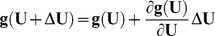
The change in displacements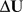 is determined iteratively such that the residual is nullified. That is
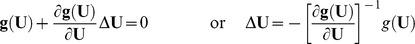
The iterations commence with **U** = **0** and the displacements are updated as **U** = **U**+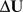. The residual *g*(**U**) is computed in each step and iterations are performed until **g**(**U**) (or 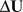) are acceptably close to **0**. The term **K** = 
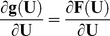
, used to compute 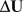 in the equation above, is the tangent stiffness matrix that is assembled using local or elemental stiffness matrices **k** obtained as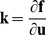.
- **Fitness evaluation.** For each model and load set, we compare the simulated reaction forces and inter-nodal distances against those measured experimentally. These errors describe how well a model explains the force and deformation of a statically loaded network system for a given set of the most informative tests. We calculate this error as follows. Let 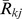 be the *j*th reaction force measured experimentally from the network for the *k*thmost informative test, and
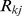
be the corresponding reaction force from a model. For *N_R_*number of output forces in a data set and for *N_DS_* such data sets, the overall functional discrepancy (in percent error), *obj_R_*, in the reaction force response is
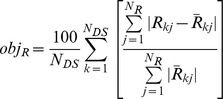
Likewise, for the network, let 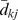 be the *j*th distance between the input and output nodes and 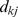 be the corresponding distance for a model in the *k*th data set. For *N_D_*distances in a data set, the overall discrepancy in the deformation response, *obj_D_*, is
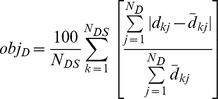
The error is computed as the arithmetic mean of the two, that is
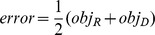
The state-of-the-art method to evaluate fitness in machine learning is to compute and compare two versions of this error: the training set (*e_training*) and cross-validation (*e_cross*) errors [e.g., 33]. The models are evolved in Stage II through the hill climber approach [33] by minimizing *e_training* = *error*. Synthesis of models is terminated when the training set error declines below a preset threshold value (models with the training set errorslower than 0.5% are regarded to explain the target system accurately), or when a steady error value is sustained for a number of evolutionary steps. Before proceeding to Stage III, each model is cross-validated to predict how well it emulates the target network using data sets different from those most informative ones used to evolve the model topologies. This fitness criterion helps to prevent over-fitting in the training set. To cross validate each model after its evolution, the following, less conservative measure, *e_cross *is used.
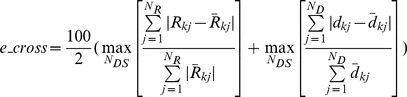
Via this error, we quantify the maximal possible functional disagreement between a model and the target network. We avoid using a similar metric for the *e_training* error since the corresponding design space is significantly non-smooth. The minimization algorithm will have difficulties in negotiating the jumps and therefore will take a significantly longer time to converge. On the other hand, the average based estimates are lower in magnitude and using those for cross validation will give a flawed impression about the match between the model and the corresponding network. In Stage III, to generate a new most informative test, we seek to maximize *error* to determine the actuation forces that cause maximal discrepancy among the models evolved in Stage II. Once such a new most informative test is found in simulation, the experiment is performed with it and a new most informative input-output force-deformation data set is added to the database with the intention of differentiating better models (those that agree with the new data set) from the worse ones (those that disagree with the new data set) to further evolve the population of models (see below). The inference process is discontinued when the maximal, preset number of the most informative data sets to which the models can be exposed, is reached.

- Although comparing the topologies of the evolved models with the known target network is a secondary (structural) fitness criterion, this is only possible with the synthetic Latex networks.

- **Model evolution.** We employ model fitness to guide the evolution of the population of models. We use a stochastic hill climber search to evolve the entire population of models [33]. The length and cross section of each string (Fig. 2a) are the free parameters that are varied to minimize the training set error, *e_training*(in iii). Stochastic changes in these free parametersare systematically performed as follows: for *c* as a free parameter (string length or cross section), *c_new_* = *c*±(*c*_U_−*c*_L_)*exp*(*R*_1_+*r R*_2_) represents its altered or mutated value about *c* (± signifies that *c_new_* can be larger or smaller than *c*) where *r* is a uniformly distributed real number ∈ [0, 1], *c*_L_and *c*_U_ are the specified lower and upper bounds on *c*, and *R*_1_ and *R*_2_ are chosen such to encourage both small and large changes. For all the experiments performed, *R*_1_ = −8 and *R*_2_ = 9 are used for which, changes in *c*, if they occur, are permitted within 3.35×10^−4^(*c*_U_−*c*_L_) and 2.72(*c*_U_−*c*_L_). If *c_new_*<*c*_L_, *c_new_* is set to *c*_L_. Otherwise, if *c_new_* exceeds *c*_U_, *c_new_* is set to *c*_U_. Changes in free parameters are performed as follows. If a uniformly distributed random number *p* represents the probability for change and if *p*<*p_rate_* (e.g., 8%), the value of the free parameter is altered. A small *p_rate_* (typically less than 15%) is chosen to prevent the hill climber algorithm from degenerating into a random search. A new model is created by altering only some free parameter values *c* of the prior model to *c_new_*. The new model is analyzed (see model analysis, **stage II**) and if it has a lower training set error, *e_training*, the new model then replaces the current model. Else, the current model is retained. To limit the computational cost and yet allow sufficient iterations/time for the models to evolve after a new most informative test is introduced, the search is continued until any one of the following termination conditions is met: (a) The training set error for a model is less than the chosen threshold (0.5%). Further evolution of this model is ceased but other models in the population are continued to evolve. (b) Before the model evolution commences in Stage II, Fig. 1, a counter *G_L_* that tracks favorable changes is set to 0. A favorable change is one that lowers the training set error of any model in the population. Another counter *G* (initially set to 0) used is such that it is incremented by 1 if none of the models improve in the training set error in an iteration of Stage II, Fig. 1. For any model, if the training set error is lowered, *G* is set to zero and *G_L_* is incremented by 1. When *G*>2*G_L_*, model evolution is stopped. It may happen that a small value of *G_L_* will lead to premature convergence to avoid which, the minimum permitted value for *G* used is 5000. (c) To permit model evolution in finite time for a newly introduced most informative test, a hard limit on the number of iterations in Stage I, Fig. 1 is set to 100,000. This limit is used for both Latex and anatomical target systems.

A few additional evolutionary strategies are also used so that the models can evolve better and faster. In the first, very small changes (<1%) in the free parameters are performed but with a higher rate (*5p_rate_*). In the second, major changes (>20%) are accomplished with a smaller rate (0.2*p_rate_*). Lastly, the worst model in the population is crossed over with a random one if the former does not exhibit improvement over a number of iterations.

After the free parameters of a chosen number of models are evolved using the informative input-output data (Stage II, Fig. 1), only those strings that become taut for at least one set of simulated tests constitute the model topology. Those that remain slack for all simulated tests do not participate in the connectivity. However, they are retained in each model as they may get taut at a later stage in the inference algorithm.

### Stage III: Evolution of the most informative tests

Once the population of models has evolved to explain the previous informative datasetsas per the termination criteria (Stage II, Fig. 1), a new most informative test is generated inStage III, Fig. 1. Whereas the fitness of the models was to explain available data, the fitness of the tests is to make the models disagree in their prediction. Tests that make the models disagree are likely to be more informative because they uncover functional differences across models.

Ideally, conducting the inference process in real time requires the availability of a large parallel computing network to evolve the models and the most informative actuation tests shortly after each new load set is added to the database. Because this is not feasible (see discussion) with manual application of loads and the need to test perishable tissue, we did the next best thing: collected the experimental data for the Latex and tendinous networks in dedicated experimental sessions—and ran the algorithm off-line using those data sets. Whenever the algorithm requests the next most informative test (found as described below), we provide the load set corresponding to the nearest neighbor (in the least squared sense) to it from the available load sets.

In our experiments, the direction of each test (input force) is fixed in the global frame, and we therefore only evolve their magnitudes. These magnitudes *F_new_* are mutated in a similar manner as the string free parameter *c_new_* described above. If *F_new_* is found to be less than *F_L_* ( = 0 N) or greater than *F_U_* ( = 5 N), the lower and upper force bounds, *F_new_* is set to *F_L_* or *F_U_* respectively. All models in Stage II, Fig. 1 are subject to these testsin simulation and the incongruity in their force-deformation response is quantified by an error *e_test* given by
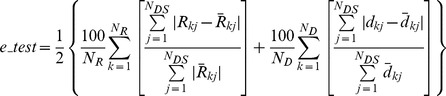
where *N_DS_* represents the number of evolved models in the population, *N_R_* is the number of reaction forces *R_kj_*, *N_D_* is the number of distances *d_kj_* between the input and output nodes, 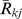 is the mean of the reaction forces over the models and 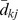 is the mean of the distances. Analteredtest replaces the previous test if the corresponding *e_test* value is higher (a larger *e_test* implies greater discrepancy suggesting that the test is more informative). Otherwise, the previous testis retained. The termination criteria for the evolution process for the most informative test (used in the subsequent experiment) are similar to those used in model evolution (Stage II, Fig. 1).

#### Experimental generation of input-output load sets

As mentioned above, conducting the inference process in real time requires the availability of a large, parallel computing network to evolve the models and the most informative actuation tests shortly after each new load set is added to the database. Because this is not feasible for this first study, we collected data for both Latex and tendinous networks in dedicated experimental sessions—and ran the algorithm off-line using those data sets. In all three experiments (inference of two Latex and one tendinous networks), we collected 72 input-output data sets in response to 72 different combinations of tests in random order to prevent any bias due to loading order (see details in Stage I below). These tests were designed to be evenly distributed in the 3D input space: The input nodes were tugged with combinations of four force levels of 1.25 N, 2.50 N, 3.75 N and 5.00 N and the resulting static force-deformation responses were measured for a total of 64 load sets (4^3^ = 64). Additionally, we interspersed eight more load sets for cross validation. Here, tensions of 1.9 N and 4.4 N (2^3^ = 8 combinations) were employed to pull each input string.

All tests were chosen to amply deform the cadaveric network and yet be conservative to not tear the collagenous cadaver tissue. Further, by introducing the tests used for cross validation uniformly between those used to generate the training set, we ensured that the cross-validation load sets were distinct from any training load set.

For all three networks, the experiments for data collection lasted about 8 hours, which is well within the time frame for studies of cadaveric tissue [37]. While it is known that the human finger tendons can uphold large forces (∼90 N) [38], a conservative range for the actuation forces was chosen for this experiment (≤5 N) to prevent rupture of tissue or tethers, or interconnections between them during repeated loading.

The collection of the 72 load sets allowed us to execute and validate the inference algorithm off-line. Whenever the algorithm requested the next most informative test, we provided the load set corresponding to the nearest neighbor (in the least squared sense) to it from the 64 training load sets. Within each cycle of the estimation-exploration algorithm, after the models were evolved, their cross-validation was performed with 8 additional load sets.

### Stage I: Seeding the load set database

In Stage I, we first picked a single load set from the experimental data at random to seed the database. This load set formed the initial database used in Stage II (Fig. 1b).

### Applying the predicted most informative tests (i.e., input forces) to the experimental synthetic Latex networks and cadaveric specimens

#### Experimental setup for the ‘AFH’ and ‘aWR’ synthetic Latex networks

**Figure 3:**
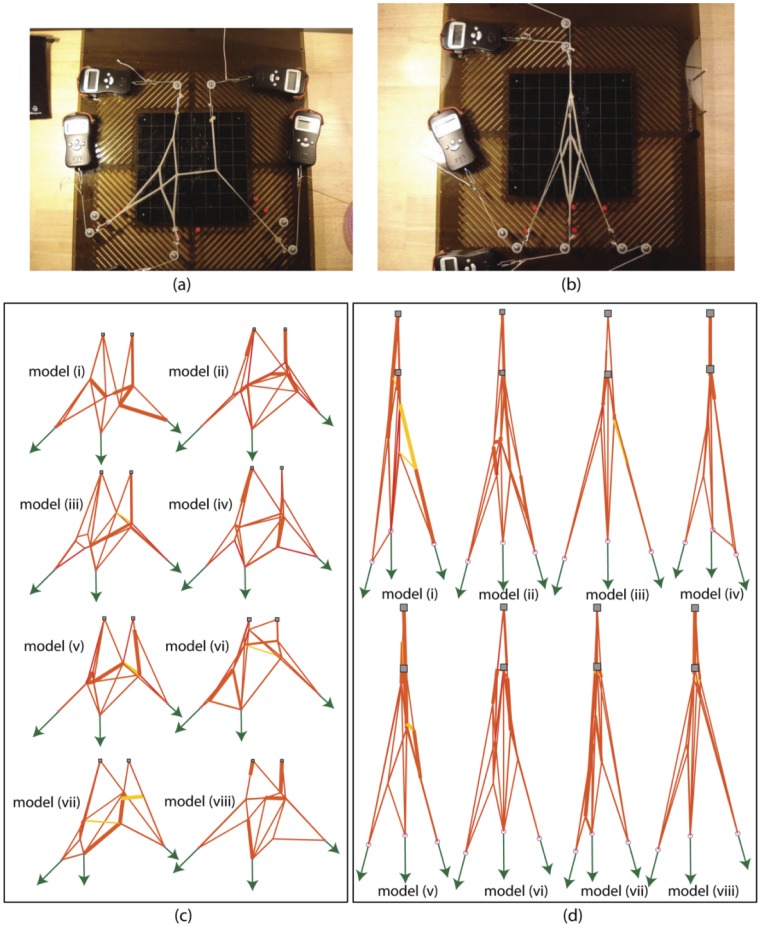
Inference of the synthetic target networks using the informative force-motion data generated from the inference process. The grounded nodes are shown using squares and the actuation forces are depicted using the arrows. Each model is evolved until they see 20 informative experimental data sets. (a) The ‘AFH’ target. (b) adapted Winslow's Rhombus or ‘aWR’ target. This is an adaptation of the Zancoli's representation [17] of the finger extensor mechanism (Fig. 4a). In the latter, the diagonal bands and lateral offshoots overlap while in this adaptation, the corresponding strings are fused. The top two grounded ports (through which the reaction forces are measured) are not interconnected. (c) Best eight models evolved through the informative data from the ‘AFH’ target. (d) Best eight models evolved through the informative data from the ‘aWR’ target. Strings colored yellow are slack in the shown equilibrium configuration. Those colored red are taut. Slack strings get taut for some other informative load set that they see during their evolution. Models (iii) in (c) and (ii) in (d) with the least cross validation errors (Table I) resemble in structural constitution with their respective targets.

We inferred two arbitrary networks: one resembling the three letters ‘AFH’ fused together (Fig. 3a), and the other representing Winslow's Rhombus or ‘aWR’ (Fig. 3b) from a synthetic Latex sheet (part SLR-040-E, 1 mm thickness, 380 mm×305 mm, Small Parts Inc.). Both networks were designed in a CAD package to fit inside a 100×100 mm square when unloaded, and cut with a computer-controlled laser cutter. Each string had a rectangular cross-section with in-plane width of 4 mm and thickness of 1 mm. The input-output nodes were shaped as perforated disks with a 5 mm diameter to prevent tearing.

Both networks had five input-output nodes, each tied to a tether attached via an alligator clip to a swiveling, grounded electronic dynamometer with a 2 *g* resolution (Fig. 3 a–b, 4c). The designated output nodes were simply grounded to dedicated dynamometers that measured the reaction forces. In contrast, the lengths of the tethers attached to the input nodes were adjusted manually to stretch or relax the network and thereby apply a specific tension to that node. The tethers were routed above the experimental bed via pulleys with adjustable locations so that (i) the network issuspended above the bed to improve the accuracy in the force readings by removing friction and stiction; and (ii) the line of action of the tests (input loads) can be adjusted. Once the loaded network attained equilibrium, the inter-nodal distances between the three input and two output nodes (6 values) were measured with a dial caliper to an accuracy of 1 mm. The deformed configuration of the network is uniquely specified by these distances.

**Figure 4:**
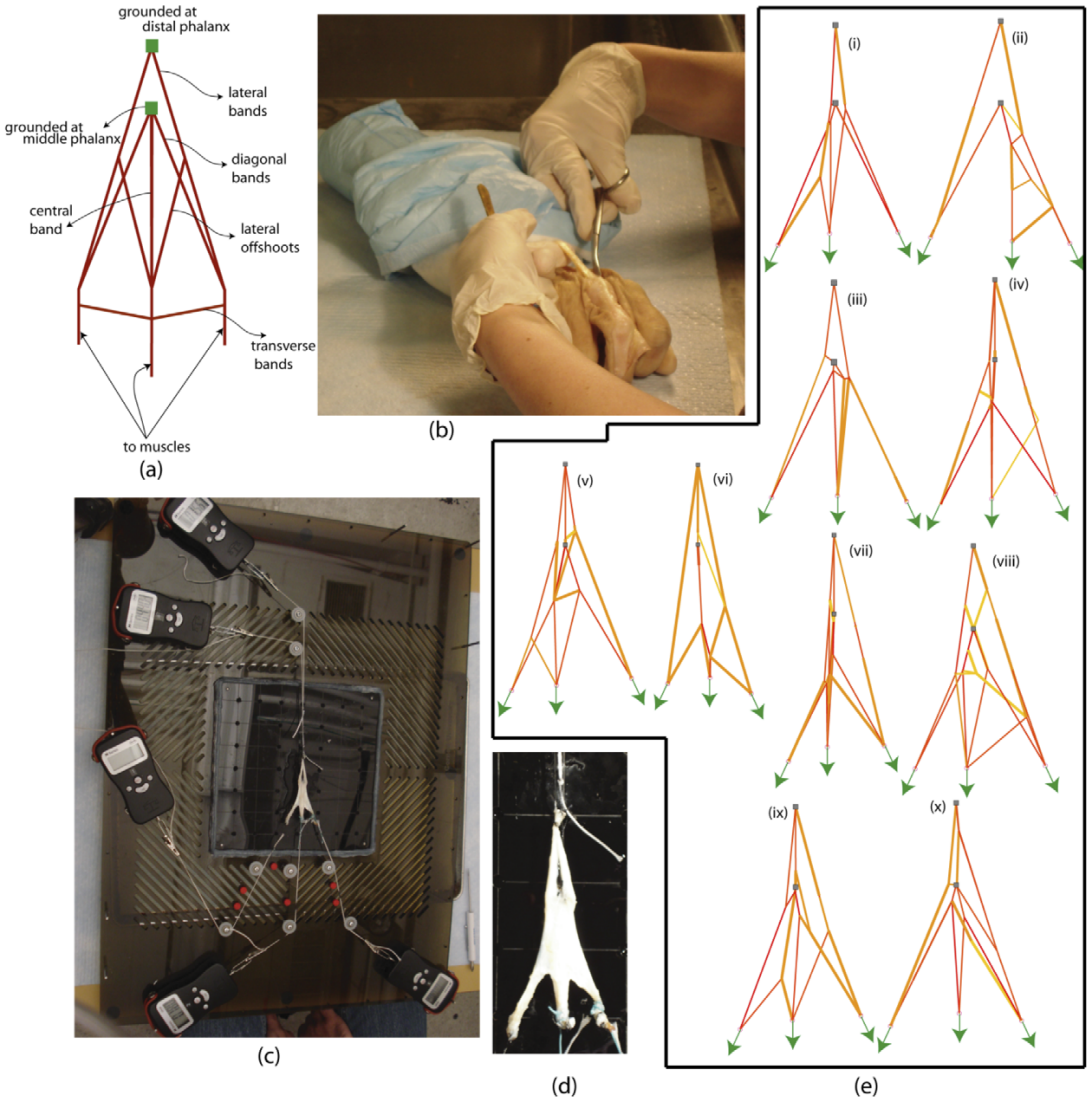
Structural inference of the finger extensor mechanism extracted from the middle finger of a human cadaver hand. (a) The interpretation by Zancoli [17] and Garcia-Elias [18] as Winslow's Rhombus is widely accepted. A characteristic of this structure is the overlap between the lateral offshoot and the diagonal band on both symmetric sides. (b) The extensor tissue was carefully extracted during the day of the experiment. (c) the tissue mounted over the experimental bed for force-motion data extraction (d) magnified view of the extensor tissue (e) ten best inferred networks that are all functionally equivalent within the training set and cross validation errors of 7.9% and 7.2% respectively (Table 2). The models are structurally diverse. Strings colored yellow are not taut in the equilibrium configuration shown. Those colored red are taut. Slack strings do become taut when some other test, used to infer the model network, is employed. Models (i), (iv), (viii) and (ix) in (e) structurally resemble with the Winslow's rhombus in (a) with notable deviations besides the presence of additional strings. In model (i), all modules according to the classical description in (a) are captured except for the left lateral offshoot which is shorter in length. In (iv), a string connects the middle and terminal slips. In (viii), the central band is replaced by a diamond. In model (ix), the left lateral offshoot does not overlap with the left diagonal band but instead, is fused with it.

When loading the ‘AFH’ and ‘aWR’ networks, the location of the input nodes was allowed to change as the networks deformed, but the lines of action were maintained constant. For the ‘AFH’ network, the directions for the tests were fixed along −135°, −90° and −45°, respectively from the horizontal at the left, center and right bottom input nodes (see Fig. 3). For the ‘aWR’ network, these directions were −105°, −90° and −75°, respectively.

#### Experimental set up for the cadaveric extensor mechanism

As in our prior cadaveric work (e.g., [37]), a fresh-frozen cadaver hand was thawed overnight and an extensor mechanism was excised from the middle finger by a practicing hand surgeon (Dr. S.S. Roach, Fig. 4b). Because it is critical to maintain the structural integrity of the tissue throughout the experiment, all load sets were collected within 8 hours of the surgical excision of the extensor mechanism. The tissue was continually kept moist using a 0.9% saline solution to prevent desiccation. Using techniques similar to those described in [37] we used a hydrophilic surgical glue [VetBond, 3 M Inc.] to attach rigid tethers (i.e., Nylon strings) to the insertion slips of the *extensor digitorum communis*, *second palmar* and *second dorsal interosseous* muscles (i.e., the three input nodes) and the two terminal slips of the extensor mechanism (i.e., the *proximal* and *distal slips*, or two output nodes). The cadaveric specimen was suspended over the experimental bed (Fig. 4c) via pulleys to eliminate stiction and friction and the input tethers were held at constant angles of −115° (left), −90° (middle) and −75° (right) from the horizontal. It is assumed that these are the approximate angles along which the three actuating muscles are orientedon the dorsum of the hand, and that the changes in these angles are negligible when the muscles coordinate to result in the finger ad-abduction.

## Results

### Inference of functional networks with informative tests

Figures 3c/d show the best eight models inferred for the ‘AFH’ and ‘aWR’ target networks, respectively. These were obtained by inferring a total of 24 models for each network (three runs, each with population of eightmodels). Each run used at most 20 informative load sets. Table 1 shows the training set (*e_training*) and cross-validation (*e_cross*) errors for each model. Model (iii) in Fig. 3c has the smallest *e_cross*and close to the smallest *e_training* errors. Visual inspection confirms its structural resemblance with the ‘AFH’ network (Fig. 3a). Model (ii) in Fig. 3d with the least cross-validation error is also topologically similar to the ‘aWR’ network. Models (iii), (vi) and (viii) that have *e_training* errors less than 3.6% are also structurally comparable to the ‘aWR’ system. However, the other four models are visually dissimilar though they are functionally similar within the *e_training*error of 3.7% and *e_cross* error of 6.4% (Table 1). Also, for *e_cross* errors less than 6.1%, models in Fig. 3c are all functionally similar to the ‘AFH’ network though topologically dissimilar. We observe structural diversity among functionally similar models for the ‘AFH’ network to within ±1.0% of *e_training *errors and ±1.3% of *e_cross* errors.

**Table 1:** Training set and cross validation errors of various models inferred through the informative data obtained from synthetic ‘AFH’ and ‘aWR’ targets (Fig. 3).

Synthetic Target	Model Number	Training set error (%)	Cross validation error (%)
Models inferred from the ‘AFH’ target (Fig. 2c)	(i)	2.9	4.2
	(ii)	3.3	5.0
	(iii)	2.8	3.6
	(iv)	2.8	4.7
	(v)	2.8	4.5
	(vi)	3.0	5.7
	(vii)	4.0	5.2
	(viii)	2.6	6.1
Models inferred from the ‘aWR’ target (Fig. 2d)	(i)	2.6	5.5
	(ii)	2.4	4.5
	(iii)	2.7	5.5
	(iv)	3.7	5.3
	(v)	2.3	5.8
	(vi)	2.6	6.1
	(vii)	3.3	5.6
	(viii)	3.6	6.4

Three independent trials are conducted to infer both the synthetic targets in Fig. 3. The mean training set and cross validation errors for the eight selected ‘AFH’ models are 3.0% and 4.9% respectively. These for eight chosen models representing the ‘aWR’ target are 2.9% and 5.6%.

Likewise, models functionally similar to the ‘aWR’ network differ in structure within *e_training *and *e_cross *errors of ±0.8% and ±1.1% respectively.

The number of functional evaluations required to infer the ‘AFH’ and ‘aWR’ networks is c. 0.7 vs. 0.5 million, respectively. The CPU times for model evolution and generation of the most informative tests on three different machines for the synthetic targets are presented in Supporting Information, Tables S1 and S2 respectively.

### Inference of functional networks with random tests

**Figure 5:**
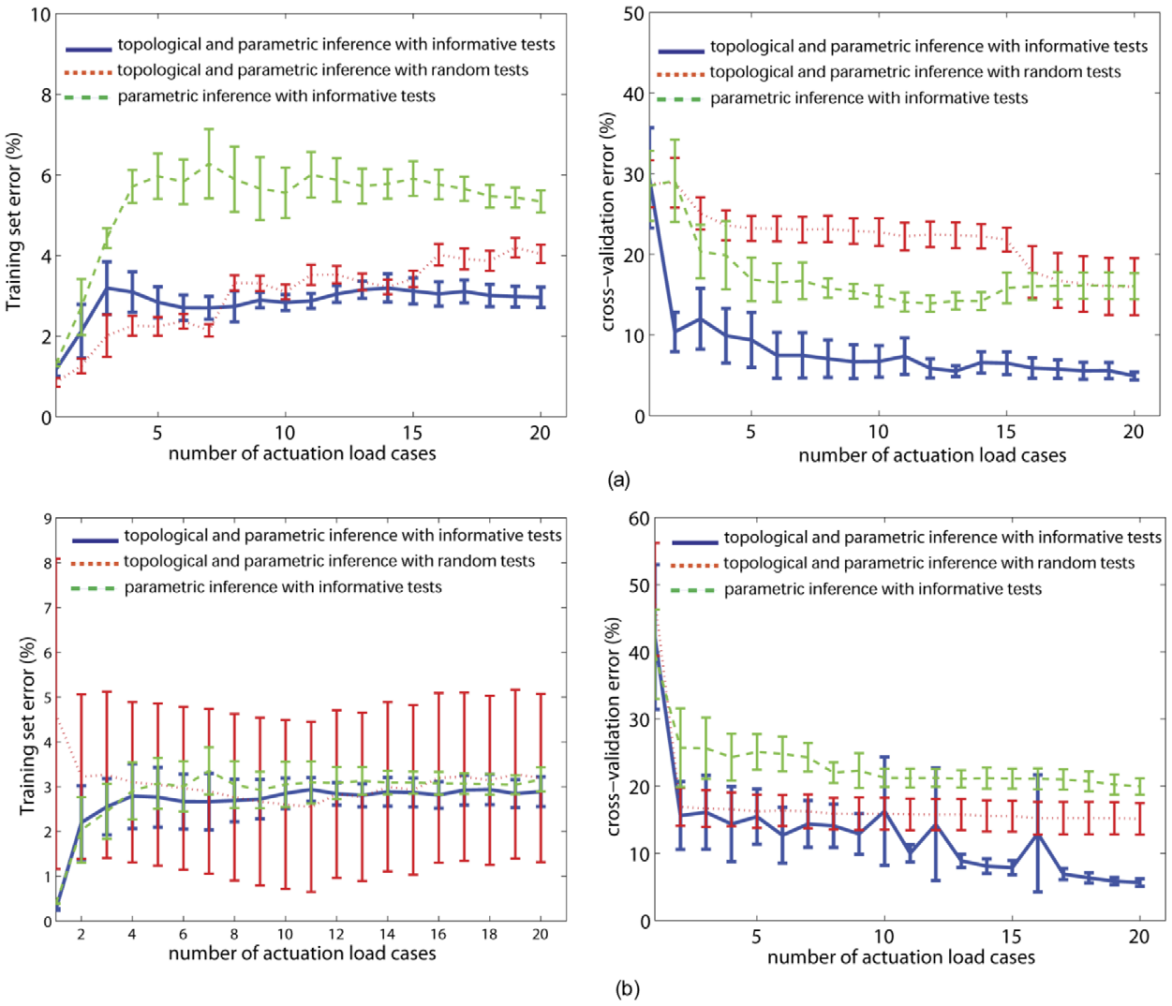
Topology matters and so does informative data generated from the inference process. Training set (left column) and cross validation (right column) errors when (a) the ‘AFH’ target is inferred and (b) when the ‘aWR’ network is inferred. Solid lines represent errors with sequential informative experimental data when both topologic and parametric inference is performed. Dotted lines correspond to cases when random experimental data is sequentially employed for inference. Dashed lines show errors for only parametric inference with basic (Fig. S2 c–d) topologies. Error bars represent standard errors across three executions of the inference processes. Topologically and parametrically inferred networks with sequentially introduced informative force-motion data are more functionally proximal to the respective targets compared to (i) when random data is used and (ii) when module/string interconnectivity is ignored during inference.

To confirm if evolving informative tests improves the inference process, we performed an additional three baseline inference runs for each target network using 20 random data sets. The *e_training* and *e_cross* errors are compared (Fig. 5), with error bars showing the standard error over three runs. The *e_training *errors with random tests are comparable to, or better than, those found using informative tests (left plots, Fig. 5). Importantly, however, the *e_cross* errors using informative tests are significantly lower for both target networks (right plots, Fig. 5).

### Comparison of parametric vs. parametric and topological inference

We also performed simple parametric fitting to infer the target networks in Figs. 3 (a) and (b) using the all-in-all (see Supporting Information, Figs. S2, c–d) topologies. When performing only parametric vs. parametric and topological inference, the numbers of free parameters used were different. In parametric fitting, only six strings were used to connect the three input nodes to the two output ones. The length and cross section of each string were evolved as in the topological inference which allowed us to use the model evolution algorithm described before. In parametric fitting, all the six strings participated by becoming taut and hence the all-in-all topology did not change.

In all cases, inferring the topology of the target network yields significantly better results (Fig. 5, green lines) than only inferring parametric values using respective all-in-alltopologies. The stated primary aim of this study is to infer the structure of hidden tendinous systems. This is important both from the evolutionary perspective where it is the actual topology that changes, and also to plan the rehabilitation and surgical repair/replacement in cases of minor/major injuries. A 3-input, 2-output system for instance the synthetic networks herein can be learnt through say, a neural network. However, such learning processes provide only a mathematical relation but lack the physical structure and insight into the complex biological systems. Thus, in our work we emphasize the need for structural models. By allowing the tendon topology to vary and comparing the data fitting results with those obtained for a presumed, primitive (all-in-all) structure, we demonstrate that performing parametric-only fitting with preconceived topologies resulting from, say, a scientist's ingenuity and insight may not necessarily lower the fitting errors. Topological inference is essential in addition to parametric-only fitting.

### Inference of a cadaver tendinous network

**Figure 6:**
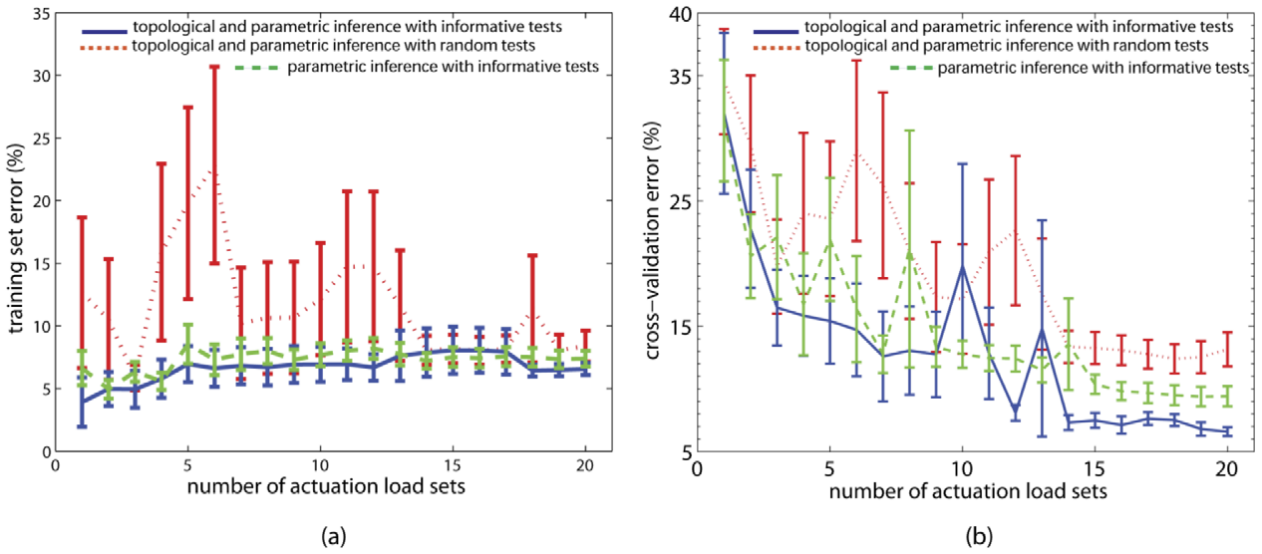
Topology matters and so does informative data generated from the inference process. Errors incurred during the inference of the finger extensor mechanism. (a) Training set errors. (b) Cross validation errors. Progression of the error values are depicted as the number of data sets are introduced for model evolution. Error values are depicted for five executions for the topologic and parametric inference using informative tests. Bars represent standard errors. Solid lines correspond to the mean error when informative data is employed in topological and parametric inference of the target. Dotted lines show mean errors when sequential random data sets are used (error bars depicted for 20 executions). Dashed lines represent mean errors (error bars depicted for 20 executions) when only parametric evolution is performed using the basic topology where in only 6 strings connect all accessible (input and output) ports and no other interconnection is allowed (Fig. S2 d). At the end of the inference process, the mean *cross validation* error when models are topologically inferred using informative tests is 6.8%, better (by about 3%) than parametric-only inference with informative tests and improved (by about 7%) compared to the topological inference with random tests. The topology chosen for parametric-only inference (Fig. S2 d) comprises most components of the Winslow's Rhombus (Fig. 4a) except for the lateral offshoots and transverse bands. We observe in Fig. 4e that some inferred models do resemble the Winslow's Rhombus structurally. The low difference (3%) in the mean *cross validation* errors suggests that the disparity between the topologically and parametrically inferred models and the parametric-only inferred models (both using informative tests) is minor but essentially topologic (we reckon this could be because of the absence of the lateral offshoots in the all-in-all topology in Fig. S2d). The difference of 7% in (b) between models inferred using informative and random tests is however, relatively significant and is expected to be larger with further improvements in the inference process (see discussion). As observed in [56], the margin gained with the estimation-exploration algorithm grows with the complexity of the problem. Additionally, one may require more number of trials (which will add to the temporal and computational cost) to achieve a certain accuracy. With the estimation-exploration algorithm, a given accuracy can be achieved faster.

Figure 4 (e) shows the ten best models from the 25 total inferred over five runs, with a population of five models per run. The *e_training *errors are below 7.9% and *e_cross* errors are below 7.2% (Table 2), which are comparable with those for the Latex networks. The mean *e_cross *error (Fig. 6 (b), solid line) converges after the models comply with the experimental information from 16 informative tests. There is no further alteration in their structures with additional informative tests. All ten models differ in topology from each other even though they are functionally similarin that they exhibit comparable *e_training *and *e_cross* errors within the limits aforementioned. Models (i), (iv), (viii) and (ix) closely resemble the Winslow's rhombus (Fig. 4 (a)), which is consistent with the findings in [17] that ignores the transverse bands. The cross-validation errors for models (i) and (iv) in Fig. 4 (e) are the lowest, with model (i) being marginally higher even though its training set error is significantly lower.

**Table 2:** Training set and cross validation errors of the ten best models inferred through the informative force-motion data obtained from the finger extensor mechanism.

	Model Number	Training set error (%)	Cross validation error (%)
Models inferred from the ‘Finger extensor mechanism’ (Fig. 4e)	(i)	3.8	6.0
	(ii)	4.0	7.0
	(iii)	5.8	6.9
	(iv)	7.1	5.8
	(v)	7.9	7.0
	(vi)	5.8	7.0
	(vii)	5.4	6.6
	(viii)	4.2	7.2
	(ix)	6.7	7.1
	(x)	5.7	7.1

Five independent inference processes are executed to infer the finger extensor mechanism. The mean training set and cross validation errors for the 10 selected models are 5.6% and 6.8%. The corresponding models are all functionally equivalent even though differing from each other in topology and parameter values.

**Figure 7:**
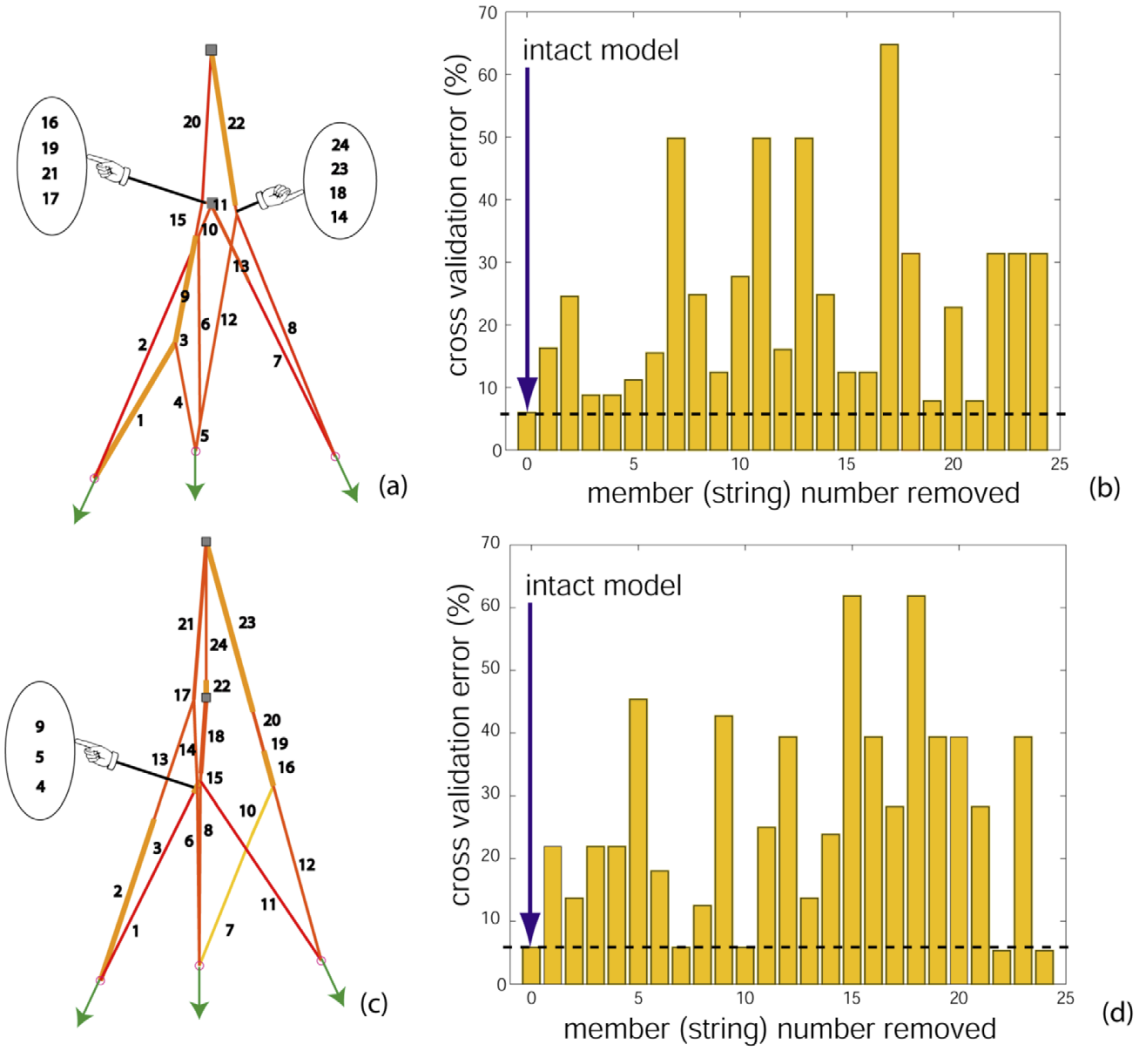
Individual strings contribute significantly to the functionality of the chosen models. (a) model (i) in Fig. 4e and (c) model (iv) in Fig. 4e with string members numbered. (b) Each string member is removed one at a time from model (i) and cross validation errors are computed. The intact model (with no string removed) exhibits the least cross validation error. (d) Each string member is removed one at a time from model (iv) and cross validation errors are computed. On removal of strings 22 or 24, the error decreases. This suggests that connection between middle and distal slips should be absent. Removing string 7 or 10 from the model in (c) does not alter the cross validation error from that of the intact model. This is because these strings do not get taut when cross validating load sets are used. Visual inspection further shows that these strings barely get taut when the model is actuated with the informative load sets used for its evolution. This suggests that strings 7 and 10 do not participate in the network which explains why the training set error for this model is larger (Table 2) compared to that of model (i) in (a).

Therefore, we chose models (i) and (iv) from Fig. 4 to further investigate the functional contribution of each member string. We eliminate one string at a time from each network and re-evaluate the *e_cross* errors. For model (i), that is shown in panel (a) in Fig. 7, the comparison of *e_cross* (Fig. 7 (b)) reveals that the intact model invariably yields the lowest *e_cross *error, demonstrating that all member strings in its topology are functionally relevant. In contrast, *e_cross* errors are lower (strings 22 and 24 removed) or the same (strings 7 and 10 removed) when several member strings are removed from model (iv) (shown in panel (d) in Fig. 7). The *e_cross* error decreases when member strings 22 and 24, which are in series and connect the grounded nodes, are removed. Such a connection does not exist in model (i) or the target network Fig. 4c/d, which suggests they are not necessary and in fact only serve to bias the loading on the grounded nodes and thus pollute the reaction forces. In contrast, the *e_cross *error remains unchanged when member strings that form the right lateral offshoot (member strings 7 and 10 connected in series) are removed. Further visual inspection of model (iv) indicates that these member strings barely get taut when the model is actuated with the informative tests used during its inference. This suggests that member strings 7 and 10 are not functionally necessary, but do not negatively affect the fitness of the network for the loading tested.

Lastly, similar to the Latex networks (Fig. 5), we report a control case (Fig. 6) with the cadaver network inference where we find that informative tests are better than random tests for both topological and parametric inference. The mean *e_training* errors are comparable in the three cases (Fig. 6 (a)), especially after the 16^th^ data set is introduced. However, the trend in Fig. 6 (b) confirms that the use of informative tests lowers the *e_cross* errors when both topological and parametric inference is performed. Importantly, we also observe that simple parametric inference with informative tests is better than the case of topological and parametric inference with random tests.

## Discussion

We demonstrate, to the best of our knowledge, the first functional inference of a complex anatomical structure using sparse experimentation. Without reverting to exploratory dissection (which is disruptive) or structural imaging (which is expensive and not necessarily informative of mechanical interactions under loading), we infer functional structure of a biological tendinous network by co-evolving the models with informative tests. We began by validating and calibrating our novel methodology using two synthetic Latex target networks, and then applied our method to the real-world problem of inference of the functional structure of the tendinous extensor mechanism tissue excised from a cadaver hand. Notwithstanding (i) the specific optimization procedure (we used a stochastic hill climber search, which are hotly debated by its supporters and retractors, but any other optimization that is suitable to the problem at hand can be used), or (ii) our current inability to run the estimation-exploration algorithm real time (we collected the experimental data for the Latex and tendinous networks in dedicated experimental sessions—and ran the algorithm off-line using those data sets), our results clearly illuminate and demonstrate several important features and concepts about this approach. These include (i) the powerful utility of a novel, general purpose predator-prey estimation-exploration algorithm for topologic and parametric inference of physical systems, and (ii) the particular functional characteristic of our test system: the extensor mechanism of the fingers whose structure and function have been debated since at least the 16^th^ century.

In this first application of the predator-prey estimation-exploration algorithm [33] for topologic and parametric inference toactual biological (cadaveric) physical systems, we demonstrate that informative tests perform better than random tests. This is critical when limited to a finite number of tests of the physical specimen, which in this case is costly and can damage the specimen by excessive testing. We define the most informative tests as those that, in simulation (Fig. 1), are evolved to maximize disagreement among the population of current models. We introduce these tests sequentially in that the population of models evolved explains the informative data available up to that point in time. We show that a small number of informative physical tests produce input-output data sets that significantly lower the cross-validation error of the resulting models. Thus, the predator-prey competition carried out in simulation findsthe most informative tests. These tests, even with minor deviations from those predicted, provide significantly useful experimental data to guide the development of the next generation of models.

We remark that, for the tendinous specimen, we needed to extract the experimental data within the first 8 hours of its excision to avoid structural and/or material degradation. Evolving the models with the most informative tests, as predicted in simulation in stage III of the inference process, was not possible with the available computational resources; the overall inference process took much more than 8 hours. Rather, we sought to access the closest possible tests that were informative, if not the most informative, from the experiments performed a priori. We further remark that for both, synthetic and biological networks, generating the most informative test was not possible with the experimental setup used (Figs. 3–4) as it required the input tensions to be achieved by pulling on the tethers manually. As the inputs were strongly interrelated, a slight manipulation of an input tether disturbed the tensions in the others. Achieving the accuracy of the recommended most informative test required much effort and was cumbersome. There were discrepancies (noise and/or measurement errors) even when efforts were made to load the network(s) with the most informative tests. While structural/material degradation was not a concern with the synthetic networks of known topologies, inference of these was performed with informative tests to verify if the latter, even when they not being the most informative, can evolve models to adequately resemble the target in structure and/or function. We show in all three cases (synthetic and biological targets) that informative tests do infer the networks, known or hidden, better than the random tests. This work is, therefore, a successful proof of concept that does demonstrate the utility of our approach and produces results that are valuable to the field of functional inference in biological systems. Based on our earlier work in [32] where we infer the Winslow's Rhombus (Fig. 4a) in simulation using the most informative force-displacement data, and supporting information (Fig. S1 c) where we infer the structure of the ‘aWR’ and ‘A’ networks in simulation but using only the most informative force data, we further expect that the most informative tests will perform better than the informative ones once better computational resources and experimental setups are available to make the overall inference process more efficient.

We also show that simultaneous topological and parametric inference yields better results than the parametric inference alone. Most system identification for biomechanical models is limited to parametric inference [38]–[40]—wherein the structure or topology of the system is chosen a priori based on expert knowledge, and parameter values are tuned to fit the experimental data. Very few studies, (e.g. [41]) have performed simultaneous inference of both the topology and parameters of anatomical systems. We show that in our experiments on synthetic and biological physical systems, the tuning of string parameters in networks with fixed topology is insufficient to minimize cross-validation errors. Importantly, cross-validation errors are a better estimate of model accuracy and generalizability because they evaluate fitness with respect to input-output data sets that were not used to train the model in the first place.

A clear distinction needs to be made between topology and parameter values. Models are assembled by exploring the space of possible combinations of building blocks (in this case, strings and nodes). A specific model topology is a specific connectivity map among a specific set of building blocks (i.e., string connectivity). The model parameters are the individual properties of each building block (i.e., length of strings). In practice, however, it is necessary to “parameterize” the topology to be able to encode (i.e., represent) it so that an algorithm can search the topological space. In our case, we defined our primordial mesh of strings (Fig. 2a) and parameterized different topologies by allowing strings to become long enough to, in practice, “disappear” because they can no longer carry tension. While this methodological distinction between topology and parameter values may be considered semantic—and therefore debatable—in practice such implementations can and do produce models with distinctively different physical structures [33]. In our case, we evolve populations of string networks with patently different number and connectivity among load-carrying strings—which assume distinct anatomical structures.

On the methodological side, several issues are key to accurate inference. These include accurate assumptions pertaining to (a) the material properties and the strain-deformation models, (b) informative experiments capturing full network functionality and interaction conditions, (c) automated experimental setup, (d) the primordial connectivity representation, and (e) the choice of the algorithm used for model evolution. If inappropriate assumptions are used, the experimental data may not be within the set of predictions of any feasible model topology or parameter values, or the candidate tests generated by the inference process may not be informative.

**Figure 8:**
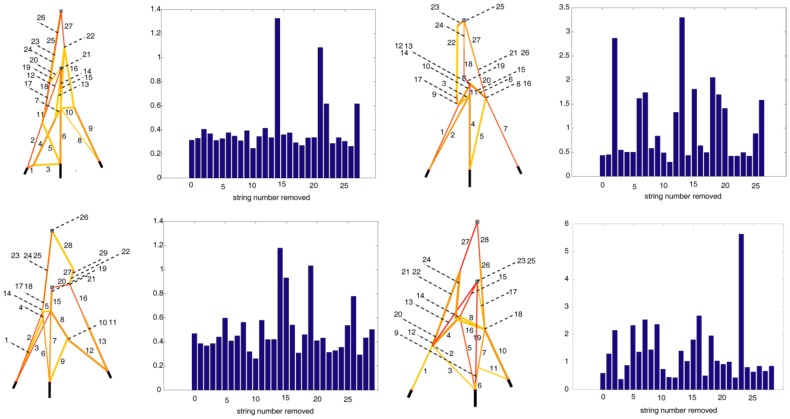
Additional information to accurately decipher the structural constitution of a finger extensor network is critical. First and third columns: Best models are evolved with linear elastic tendon properties (E = 1 GPa). The models should emulate the informative experimental data from the cadaver hand. String numbers shown with the evolved networks. Second and fourth columns: Cross validation errors (×100 for percentage) for corresponding models on the left. The first bar represents the error when the corresponding network on the left is intact. The following bars depict the error when the strings are snapped one at a time. The errors for the intact models are equal to or greater than 30%. From each model, elimination of certain strings lowers the cross validation errors suggesting that the intact models do not depict the functionality of the actual extensor mechanism accurately. If erroneous postulations are employed, the experimental data obtained by the inference process may not be informative after all.

The inference of the extensor mechanism was also performed assuming constant elastic modulus (1 GPa) as opposed to nonlinear stress-strain relationship for tendons used from [34] to evolve the models in Fig. 4. The *e_cross *errors of the resulting models were about 30% (Fig. 8), much higher than the *e_cross* errors (Table 2) for the models in Fig. 4. Inaccurate assumptions for the tendon material properties led to the evolution of models whose functionality was not in agreement with that of the target extensor mechanism. Alternatively, while it is possible to evolve the material properties as well along with the models, approximating the form and range of the stress-strain relation (e.g., exponential, transcendental, linear) may be difficult. Use of a different set of material properties can lead to either (i) erroneous structural and parametric predictions with high *e-training* or *e_cross *errors for the models, or (ii) an alternate set of functionally similar models that explain the network functionality reasonably well.

All the string networks were inferred here using the large deformation-small strain assumption. Models may also be evolved using the Green-Lagrange large-strain [35]–[36] theory. In case of the tendinous tissue, the anatomical extensor mechanism did not exhibit significant deformationand thus, the small-strain assumption was suitable.

We inferred the hand extensor mechanism by considering only a part of its overall functionality. We were limited to testing the anatomical specimen as it lay flat on a hydrated surface, as opposed to wrapped over finger joints. Even so, all string modules, as described in the classical Winslow's model (Fig. 4 (a)), were nearly captured in some models.

To reproduce the most informative tests recommended by Stage III of the estimation exploration algorithm, the setup should be fully automated and computer-controlled. Independent motors should be mounted with the respective input tethers wrapped tightly around them to control the tensions. In addition to using digital force scales, force sensors (e.g., strain gages) should also be used to control the motor rotations via a feedback loop. Achieving the most informative test with the automated setup (as opposed to manual) will be less painstaking and more accurate.

The Latex and biological specimens are inferred using the string model representations shown in Supporting Information, Fig. S2. Both the number and interconnectivity between the strings in that primordial network can influence model evolution. Here, we allow the strings to be both overlapping and tightly connected (small circles in meshes in Fig. S2). This initial mesh is chosen to facilitate the model(s) inferred from the finger extensor mechanism to assume the form of Winslow's Rhombus. A primordial mesh with no overlapping strings can yield a different set of models. Factually, however, the extensor mechanism is a sheath of collagen fibers. Using two-dimensional parameterizations (e.g., rectangular/hexagonal cells to represent the primordial mesh) canhelp yield more topologically diverse models.

All factors mentioned above, and the noise involved in experimental data influence the landscape of the objective function. In view of this, functionally similar but topologically and parametrically diverse models obtained through the Random Mutation Hill Climber (RMHC, a variant of Genetic Algorithm that employs only mutation) could all be local optima existing very close to the global one in the design space. Due to the noise present in the data, it is not expected for a global optimum to have significantly lower *e_training* and *e_cross* errors. As an aside, we show that the RMHC is capable of finding a close to global optimum for smooth functions (see supporting information, Fig. S3), even when the design space is infested with numerous local optima, if adequate computational resources are employed. We also show that alternative, classical optimization algorithms often converge to a local minimum. Further, they will not be able to negotiate the discontinuities in the design space such as those in our problem which correspond to cases wherein nonlinear analyses do not converge for candidate models.

One of the goals was to confirm whether the Winslow's Rhombus (Fig. 4 a) is an accurate string representation of the hand extensor mechanism. On performing parametric only fitting with this representation (but without transverse bands) as the primordial mesh and informative data generated using the predator-prey approach, we found that the mean *e_training* and *e_cross *errors (supporting information, Fig. S4) were comparable to those of the topologically and parametrically inferred models (Fig. 4 (e)) in Fig. 6. This suggests that the Winslow's Rhombus could belong to the same family as these functionally similar models. As detailed later, additional information may be necessary to extract a true global model.

The predator-prey approach is an optimal experiment design (OED) method wherein through competition, most informative tests (optimal sample points) are generated to evolve the best models that explain these tests. However, this approach is unlike other OED methods, e.g., D-optimal, L-optimal and minimax-optimal wherein model parameters (or their functions) are determined by minimizing, for instance, generalized variance. In Bayesian type OEDs, prior information on model parameters is assumed.

With regard to the study of complex biomechanical structures by anatomists, biomechanists and biologists, most previous work has naturally focused on inferring the structure of the tendinous networks via dissection or imaging. In contrast, we interrogate biological networks through a non-invasive computational machine learning procedure. Invasive techniques may damage the tissue, while imaging methods may miss critical functional interactions (e.g., seen only under specific loading conditions). Our non-destructive inference method yields both topological and parametric information. For example, the specific number and lengths of the tendinous members of the extensor mechanism affect the distribution of tension to the finger joints [19], [41]. We suggested before that the interpretation by Zancolli [17] and Garcia-Elias [18] of the hand extensor mechanism as Winslow's Rhombus is partly correct. We also illustrated that functionally similar models that have different string connectivity can exist to explain the functionality of the extensor mechanism. We reckon that additional information may be necessary to identify the details of structurally diverse models that exhibit similar functionality.

This raises the important issues of uniqueness and observability, which are central to computational model inference—and critical in the context of biological populations that naturally exhibit anatomical variability. Some 2D sub-topologies can be equivalent to each other under certain parameter and loading sets [32]. The load transfer patterns in these substructures can be similar despite their structural diversity. Due to these equivalencies, some substructures in a model can be transformed into one another resulting in a number of similar models. Consequently, multiple local optima as opposed to single global optimum may exist in the design space.

From the computational perspective, our use of populations of models forrandom mutation based hill climber search is very much conducive to the maintenance of model diversity (i.e., alternative hypotheses) to understand the uniqueness and observability of model topologies. This allows our search in this large dimensional space to proceed along multiple alternative paths that do not favor any particular local minimum. In all of our results with synthetic or anatomical networks, we find families of solutions: multiple different, yet functionally similar, topologies. This suggests that (i) additional data are necessary to further constrain the search, (ii) that different functional domains (such as deformation during finger flexion) are necessary to make the differences across various models observable, or (iii) that there are indeed functionally similar implementations for the domain of behavior that we studied (load transmission in this case). The latter idea is quite intriguing from the evolutionary perspective as it agrees with the well-documented natural variability in the gross anatomical structure of the extensor mechanism across humans [19]. It suggests that, for the types of anatomical structures achievable with collagen fibers, anatomical variability in human population may not be functionally detrimental, and may in fact enable a wider variety of adaptations in future generations. Thus, the popular representation of Winslow's Rhombus (Fig. 4 (a)) may no longer be considered a uniquely valid or accurate representation of the extensor mechanism. Furthermore, we observe that only a few models inferred from the human extensor mechanism concur with its classical description. While Winslow's anatomy book [16, pub. 1732] has no illustrations, the first graphical string model depiction of the extensor mechanism is by Zancolli [17] and An et al [38] wherein the tendinous network is suggested to have crossover tendons that slide past each other. In most models obtained with our experimental loading conditions, sliding crossover tendons are seldom observed and they do not grant particularly higher fitness—even though the primordial mesh was specifically designed to allow for them. In our detailed dissections of the extensor mechanism as well, crossover tendons were not clearly and independently observed.

From the clinical perspective, damage to this network can cause severe dysfunction of manipulation (e.g., mallet finger, swan-neck or boutonnière deformity [42]–[48]), which can significantly affect a person's quality of life. Both non-operative and surgical methods [49 for a brief overview, 50–55] are reported following which subjects undergo rigorous, rehabilitative tendon gliding exercises [49]. Through accurate structural and parametric prediction of the target biological network with the aim to determine sources of injuries and/or deformities specific to the patients, or the classification of patients into structurally/functionally similar subgroups, our methods and results can help plan surgical/corrective strategies more effectively (e.g., multiple trial-and-error procedures can be avoided) requiring less rigorous and more cost effective rehabilitative follow-ups. By allowing the inference of functional interactions in musculoskeletal systems, they are also relevant to the understanding of the functional adaptations that led to the evolution of the modern human hand and body.

## Supporting Information

Figure S1 We establish here the information mode – whether force or motion (distances between the accessible ports) data, or both will be required through a data set for inferring the functionality and physical manifestation of the target network. We illustrate by inferring two ‘simulated’ targets, the ‘A’ network and a minor adaptation of the Winslow's Rhombus (Fig. S1 a, b), that the use of force information alone will be inadequate. We perform model evolution using a sequence of the informative data sets generated using the inference procedure described in the paper. The two evolved ‘A’ models and that representing the Winslow's rhombus are shown in Fig. S1 (c) top, bottom. Only the force information is employed to minimize the training set error for model synthesis. Through visual comparison, we observe that even when the respective topologies are captured, there exist significant discrepancies in distances between the accessible ports when comparing the models with their respective targets. Maintaining these distances is an essential functional requirement for which reason, it is important to record, compare and minimize the differences in the respective inter-nodal distances as well when evolving the models. (a)–(b) Two simulated target networks (a: the ‘A’ target; b: adaptation of the Winslow's rhombus) employed to illustrate that the experimental data containing the force information alone is not adequate for accurate topological/parametric inference. (top) two models inferred from the ‘A’ target data (15 informative force data sets were used) and (bottom) model inferred through simulated experiments from the adapted Winslow's target network (20 informative force data sets were used). The models evolved using the informative data are topologically very similar to the respective targets. By using only the force information, the inter-nodal distances between the actuation and fixed (shown as squares) ports are not captured from the targets.
(TIFF)

Figure S2Respective representative member string fabrics used to infer the ‘AFH’ and ‘aWR’ targets (Fig. 2a–b). In (a), 36 member strings are used. Length bounds are kept as 0.01 mm and 100 mm. Cross-sections are evolved within [0.01 5] mm^2^. In (b) 54 member strings are employed. Length bounds used are [0.01 70] mm and cross sections are evolved within the same limits. (c)–(d) present respective basic topologies used to parametrically fit the informative force-motion experimental data from the ‘AFH’ and ‘aWR’ targets. (e) Representative member string fabric used to infer the finger extensor tendinous network. Seventy-one member strings are used. For each member string, lengths were evolved with the limits of 0.01 mm and 33 mm respectively while their cross sections were bound within [0.01 11] mm^2^. The upper bounds on member string lengths and cross sections were chosen based on the overall length of the tissue (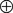 50 mm) and estimated maximum thickness of one of the input tendons (3.6–3.8 mm diameter).(TIFF)

Figure S3 The Random Mutation Hill Climber Algorithm (RMHC) implemented in the predator-prey strategy is capable of yielding solutions that are close to a global optimum if adequate computational resources are provided. Minimization of *f*(*x*) = (1+*x* ^2^)(1−½ sin(4*x*)), that has many local optima, is performed using Sequential Quadratic Programming (SQP) and RMHC. Small squares show the initial guesses *igc_i_* used by the SQP algorithm to converge to local optima shown by *oc_i_*. If the initial guesses are far from the global optimum, classical algorithms may not converge to the latter. The large squares illustrate the initial guesses *igs_i_* used by the RMHC to converge to the global optimum *os_i_*. The three attempts took 3392, 2190 and 3391 iterations respectively.
(TIFF)

Figure S4 Training and cross-validation errors when the estimation-exploration algorithm is performed using the Winslow's Rhombus (Fig. 4a) without the transverse bands as the primordial mesh. Error values are depicted for 10 best models for the topologic and parametric inference using informative tests. Bars represent standard errors. At the end of the inference process, the mean *training set* and *cross validation* errors are 8.54% and 8.44% respectively.
(TIFF)

Table S1 CPU Time (seconds) for model evolution (8 models) and generation of the most informative test for the ‘AFH’ synthetic target.
(DOCX)

Table S2 CPU Time (seconds) for model evolution (8 models) and generation of the most informative test for the ‘aWR’ synthetic target.
(DOCX)
