# Supplementary figures and images for: Functional Inference of Complex Anatomical Tendinous Networks at a Macroscopic Scale via Sparse Experimentation
Source: PLoS Comput Biol. 2012 Nov 8;8(11):e1002751. doi: 10.1371/journal.pcbi.1002751 (PMC3493461; doi:10.1371/journal.pcbi.1002751)

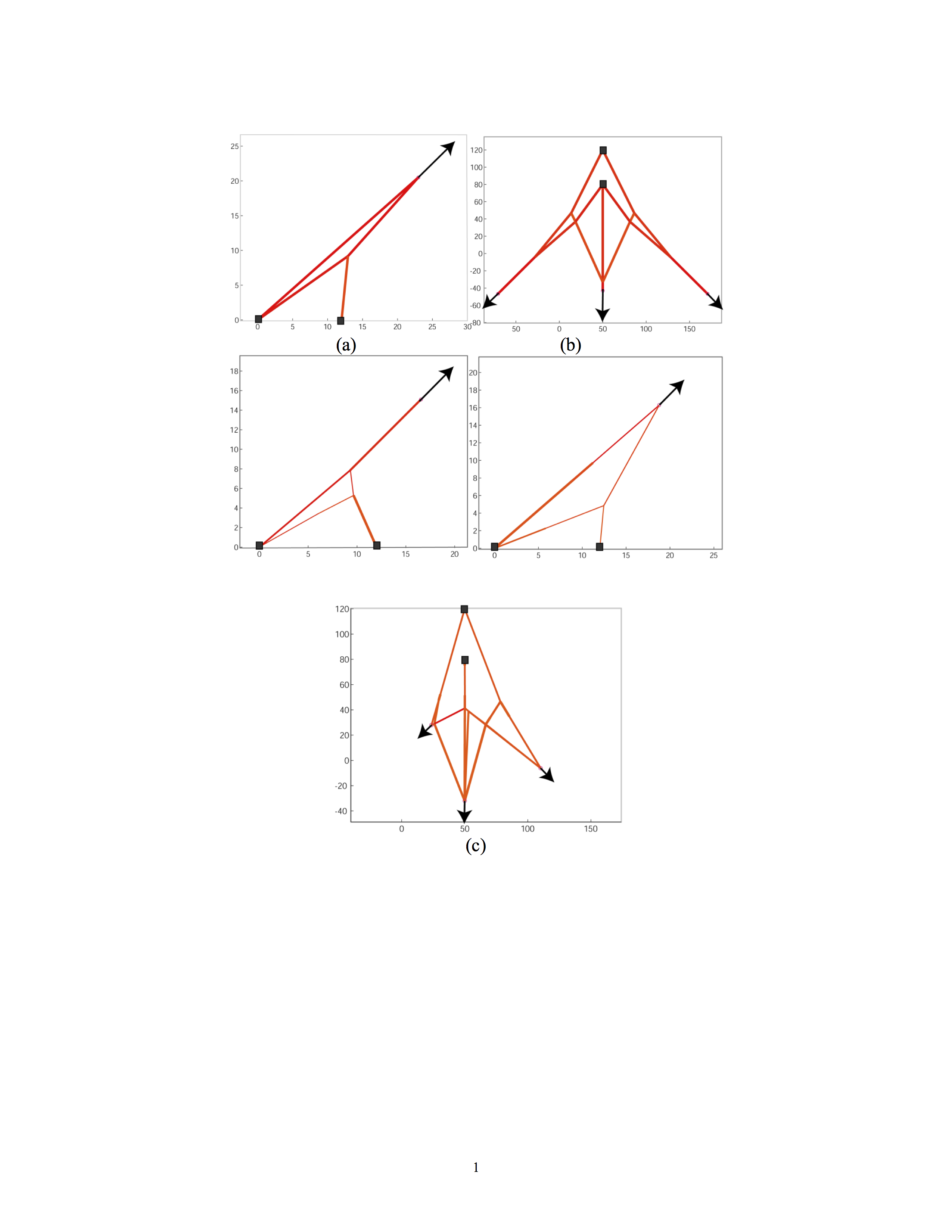

Supplement: Figure S1 — We establish here the information mode – whether force or motion (distances between the accessible ports) data, or both will be required through a data set for inferring the functionality and physical manifestation of the target network. We illustrate by inferring two ‘simulated’ targets, the ‘A’ network and a minor adaptation of the Winslow's Rhombus (Fig. S1 a, b), that the use of force information alone will be inadequate. We perform model evolution using a sequence of the informative data sets generated using the inference procedure described in the paper. The two evolved ‘A’ models and that representing the Winslow's rhombus are shown in Fig. S1 (c) top, bottom. Only the force information is employed to minimize the training set error for model synthesis. Through visual comparison, we observe that even when the respective topologies are captured, there exist significant discrepancies in distances between the accessible ports when comparing the models with their respective targets. Maintaining these distances is an essential functional requirement for which reason, it is important to record, compare and minimize the differences in the respective inter-nodal distances as well when evolving the models. (a)–(b) Two simulated target networks (a: the ‘A’ target; b: adaptation of the Winslow's rhombus) employed to illustrate that the experimental data containing the force information alone is not adequate for accurate topological/parametric inference. (top) two models inferred from the ‘A’ target data (15 informative force data sets were used) and (bottom) model inferred through simulated experiments from the adapted Winslow's target network (20 informative force data sets were used). The models evolved using the informative data are topologically very similar to the respective targets. By using only the force information, the inter-nodal distances between the actuation and fixed (shown as squares) ports are not captured from the targets. (TIFF) [file pcbi.1002751.s001.tiff]

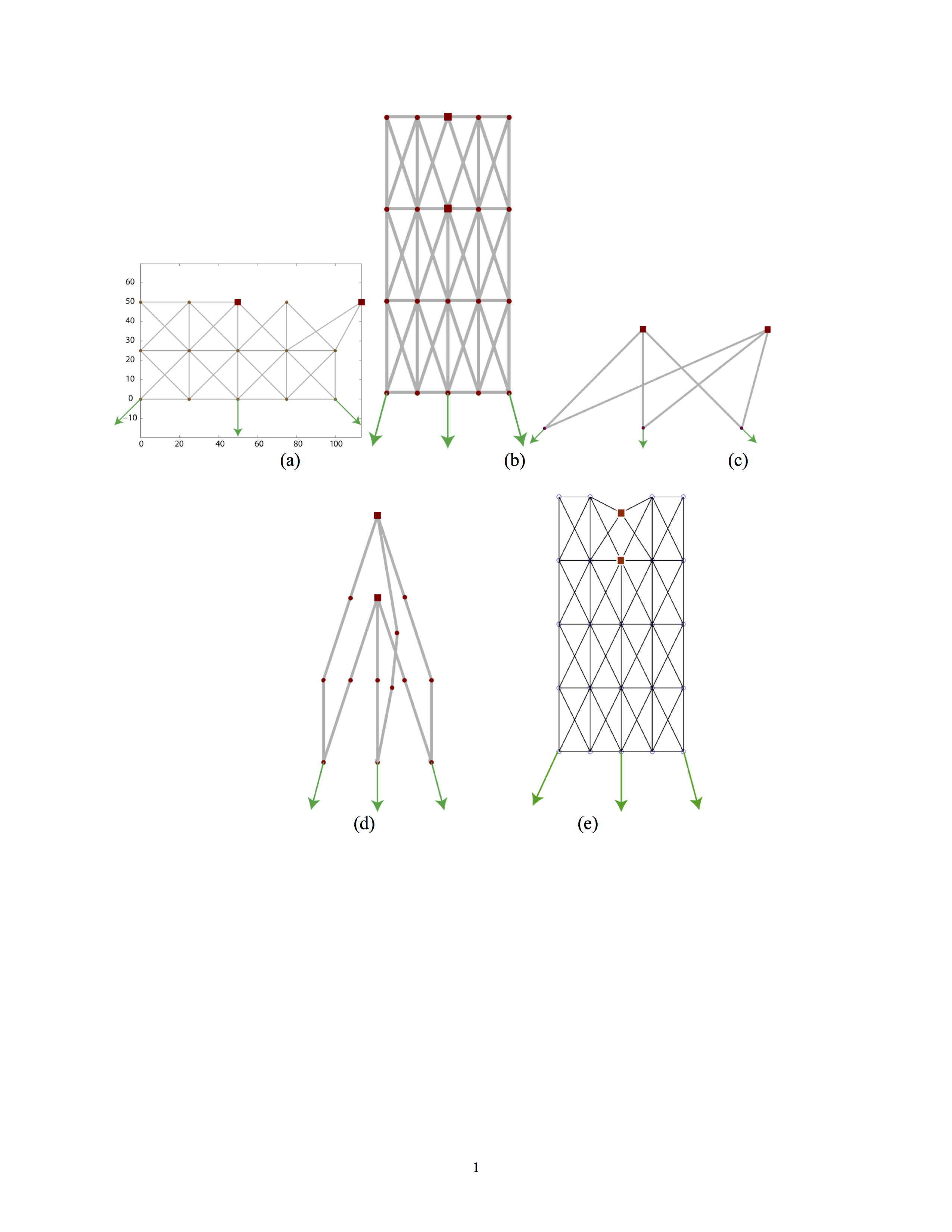

Supplement: Figure S2 — Respective representative member string fabrics used to infer the ‘AFH’ and ‘aWR’ targets (Fig. 2a–b). In (a), 36 member strings are used. Length bounds are kept as 0.01 mm and 100 mm. Cross-sections are evolved within [0.01 5] mm2. In (b) 54 member strings are employed. Length bounds used are [0.01 70] mm and cross sections are evolved within the same limits. (c)–(d) present respective basic topologies used to parametrically fit the informative force-motion experimental data from the ‘AFH’ and ‘aWR’ targets. (e) Representative member string fabric used to infer the finger extensor tendinous network. Seventy-one member strings are used. For each member string, lengths were evolved with the limits of 0.01 mm and 33 mm respectively while their cross sections were bound within [0.01 11] mm2. The upper bounds on member string lengths and cross sections were chosen based on the overall length of the tissue ( 50 mm) and estimated maximum thickness of one of the input tendons (3.6–3.8 mm diameter). (TIFF) [file pcbi.1002751.s002.tiff]

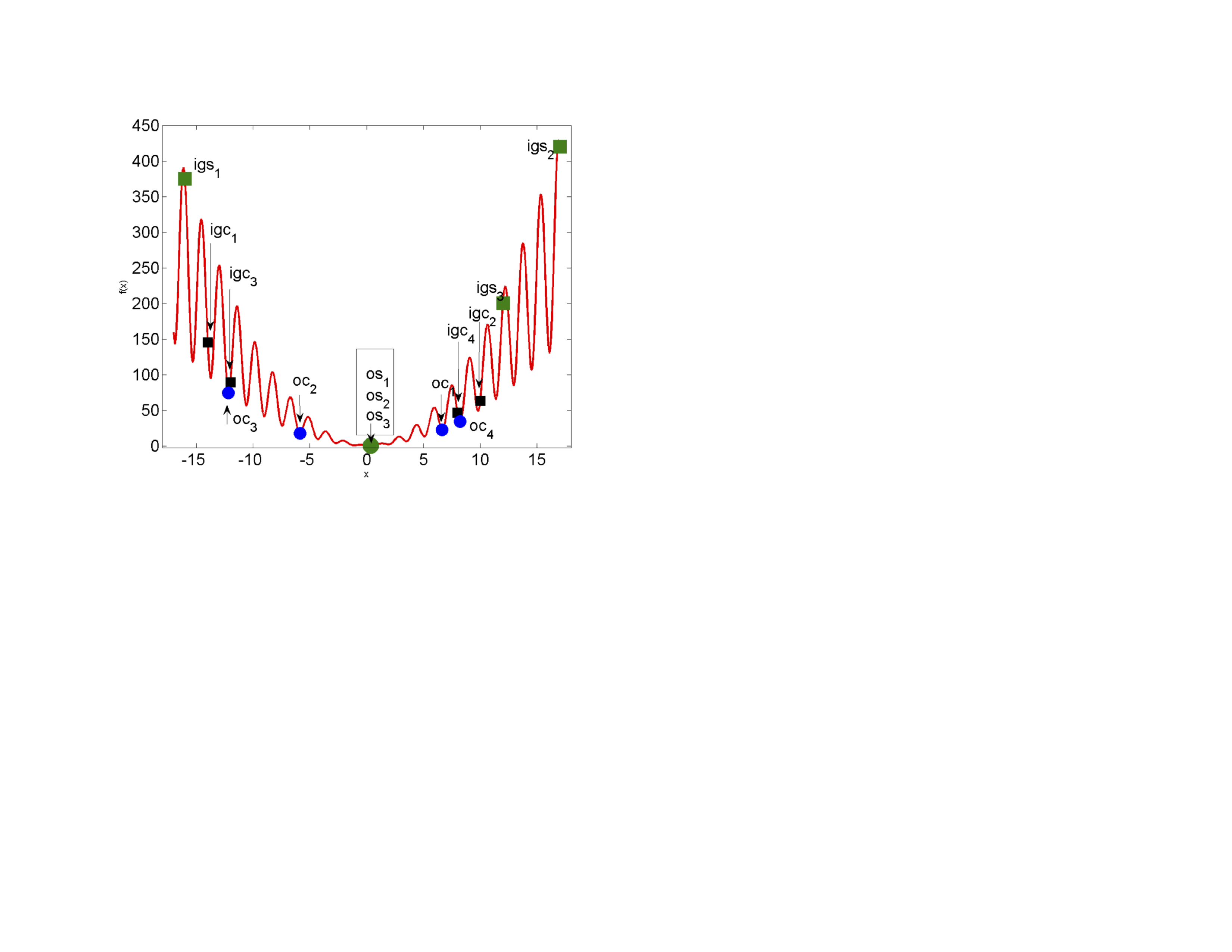

Supplement: Figure S3 — The Random Mutation Hill Climber Algorithm (RMHC) implemented in the predator-prey strategy is capable of yielding solutions that are close to a global optimum if adequate computational resources are provided. Minimization of f(x) = (1+x 2)(1−½ sin(4x)), that has many local optima, is performed using Sequential Quadratic Programming (SQP) and RMHC. Small squares show the initial guesses igci used by the SQP algorithm to converge to local optima shown by oci. If the initial guesses are far from the global optimum, classical algorithms may not converge to the latter. The large squares illustrate the initial guesses igsi used by the RMHC to converge to the global optimum osi. The three attempts took 3392, 2190 and 3391 iterations respectively. (TIFF) [file pcbi.1002751.s003.tiff]

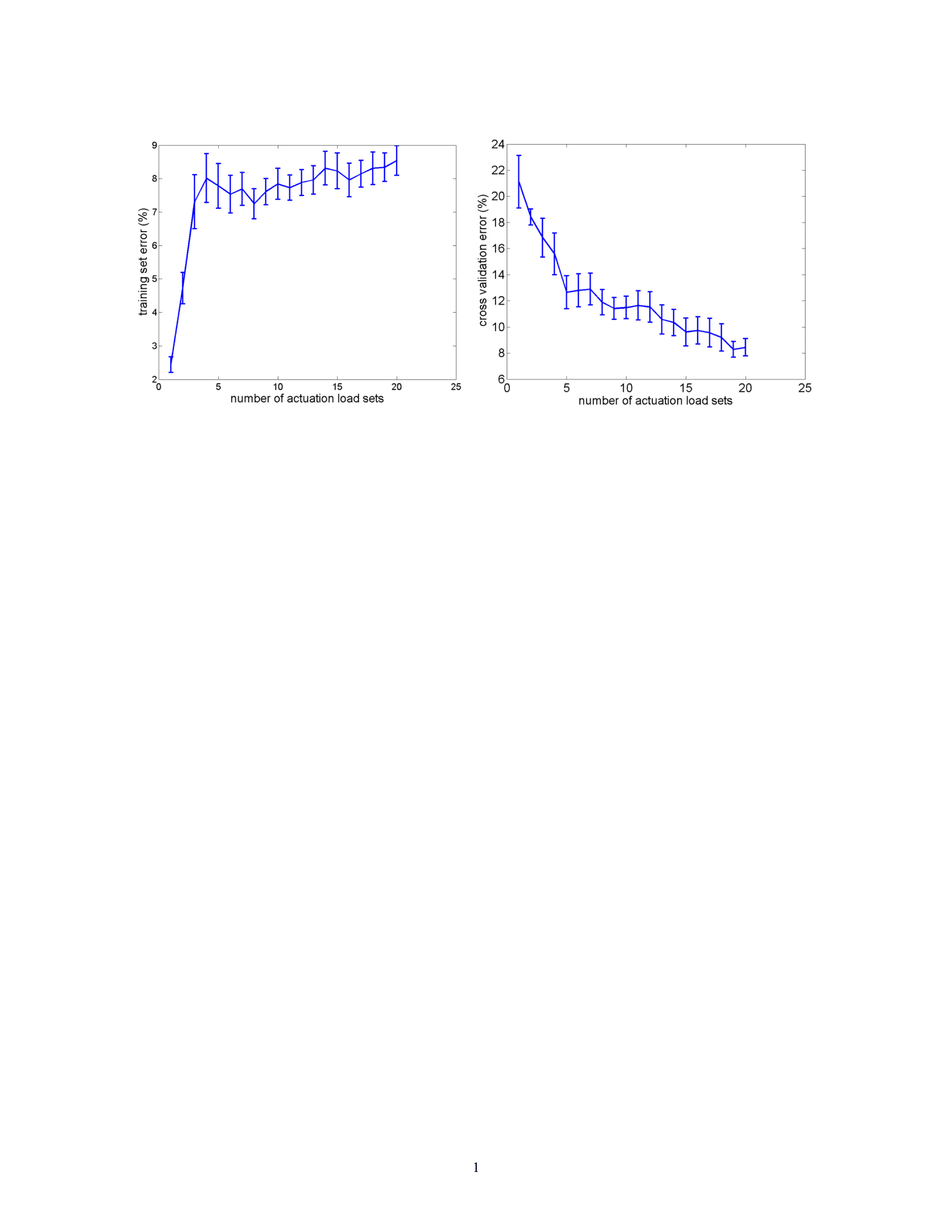

Supplement: Figure S4 — Training and cross-validation errors when the estimation-exploration algorithm is performed using the Winslow's Rhombus (Fig. 4a) without the transverse bands as the primordial mesh. Error values are depicted for 10 best models for the topologic and parametric inference using informative tests. Bars represent standard errors. At the end of the inference process, the mean training set and cross validation errors are 8.54% and 8.44% respectively. (TIFF) [file pcbi.1002751.s004.tiff]
