# Supplementary material for: Functional Inference of Complex Anatomical Tendinous Networks at a Macroscopic Scale via Sparse Experimentation
Source: PLoS Comput Biol. 2012 Nov 8;8(11):e1002751. doi: 10.1371/journal.pcbi.1002751 (PMC3493461; doi:10.1371/journal.pcbi.1002751)
Supplement: Table S1 — CPU Time (seconds) for model evolution (8 models) and generation of the most informative test for the ‘AFH’ synthetic target. (DOCX) [file pcbi.1002751.s005.docx]

| Number of tests | Machine | | | | | |
| --- | --- | --- | --- | --- | --- | --- |
|  | Machine I  Intel Pentium (4) CPU, 3.2 GHz, 4 GB RAM | | Machine II  Intel Core i7 CPU 2600 @ 3.14 GHz  16 GB RAM | | Machine III  Intel Core CPU 6420 @ 2.13 GHz  4 GB RAM | |
|  | Model evolution | Evolution of the most informative test | Model evolution | Evolution of the most informative test | Model evolution | Evolution of the most informative test |
| 1 | 804.73 | 819.02 | 547.29 | 366.35 | 433.0040 | 460.2950 |
| 2 | 398.67 | 1604.20 | 59.88 | 201.56 | 90.82 | 8006.28 |
| 3 | 769.06 | 4348.06 | 482.40 | 1519.05 | 529.40 | 1576.07 |
| 4 | 2833.27 | 4255.12 | 162.49 | 460.76 | 84.56 | 453.46 |
| 5 | 954.71 | 3653.62 | 234.26 | 624.75 | 88.10 | 3304.67 |
| 6 | 1200.94 | 3532.42 | 297.13 | 353.96 | 99.32 | 412.76 |
| 7 | 1256.60 | 3327.16 | 412.84 | 669.53 | 170.42 | 451.30 |
| 8 | 1180.47 | 3060.77 | 353.98 | 515.19 | 215.87 | 17473.01 |
| 9 | 1269.90 | 2669.82 | 494.28 | 575.53 | 401.85 | 1190.97 |
| 10 | 1389.15 | 2852.68 | 544.48 | 590.83 | 260.52 | 557.27 |
| 11 | 1607.09 | 3393.20 | 544.16 | 563.78 | 626.35 | 1159.01 |
| 12 | 1794.31 | 4131.14 | 267.18 | 315.52 | 326.80 | 568.74 |
| 13 | 1944.84 | 2804.29 | 314.10 | 380.73 | 327.62 | 1124.82 |
| 14 | 1875.73 | 3166.60 | 340.70 | 481.12 | 377.81 | 453.18 |
| 15 | 2246.25 | 2614.57 | 276.38 | 199.85 | 268.84 | 441.09 |
| 16 | 2215.39 | 2621.05 | 106.97 | 182.31 | 278.13 | 22251.24 |
| 17 | 2506.66 | 2585.95 | 206.15 | 290.44 | 350.11 | 501.63 |
| 18 | 3107.82 | 2628.25 | 167.85 | 183.78 | 357.39 | 454.63 |
| 19 | 3174.14 | 2468.46 | 238.94 | 221.44 | 377.33 | 533.75 |
| 20 | 3472.46 | 2668.63 | 297.00 | 336.55 | 413.25 | 478.86 |
