# Supplementary material for: Functional Inference of Complex Anatomical Tendinous Networks at a Macroscopic Scale via Sparse Experimentation
Source: PLoS Comput Biol. 2012 Nov 8;8(11):e1002751. doi: 10.1371/journal.pcbi.1002751 (PMC3493461; doi:10.1371/journal.pcbi.1002751)
Supplement: Table S2 — CPU Time (seconds) for model evolution (8 models) and generation of the most informative test for the ‘aWR’ synthetic target. (DOCX) [file pcbi.1002751.s006.docx]

| Number of tests | Machine | | | | | |
| --- | --- | --- | --- | --- | --- | --- |
|  | Machine I  Intel Pentium (4) CPU, 3.2 GHz, 4 GB RAM | | Machine II  Intel Core i7 CPU 2600 @ 3.14 GHz  16 GB RAM | | Machine III  Intel Core CPU 6420 @ 2.13 GHz  4 GB RAM | |
|  | Model evolution | Evolution of the most informative test | Model evolution | Evolution of the most informative test | Model evolution | Evolution of the most informative test |
| 1 | 705.3170 | 3743.3450 | 312.5210 | 2004.8690 | 804.6220 | 7586.9130 |
| 2 | 287.7190 | 698.7970 | 678.9440 | 993.0980 | 456.1610 | 892.3980 |
| 3 | 3122.0630 | 5215.7660 | 127.1870 | 327.9750 | 1017.6830 | 2109.5890 |
| 4 | 1210.1250 | 4351.5310 | 56.8780 | 624.0480 | 176.9740 | 1047.0480 |
| 5 | 2046.7500 | 2460.6560 | 77.6720 | 264.4050 | 136.0230 | 1622.3700 |
| 6 | 1838.0470 | 1319.5470 | 338.3800 | 357.1160 | 324.9780 | 915.3910 |
| 7 | 1909.0630 | 1323.5310 | 241.7070 | 293.7800 | 665.9610 | 1494.4360 |
| 8 | 2983.1720 | 1343.2500 | 240.5680 | 1147.0540 | 11996.0370 | 3417.4950 |
| 9 | 594.5470 | 745.9840 | 399.1890 | 404.7270 | 1296.7830 | 7821.9790 |
| 10 | 1751.1560 | 2905.6870 | 161.2580 | 336.6800 | 3064.5510 | 2739.0830 |
| 11 | 3680.5800 | 4480.4220 | 312.8590 | 1847.9170 | 2588.3120 | 1414.5790 |
| 12 | 3225.3850 | 1986.4450 | 1287.3770 | 1720.8230 | 3271.1360 | 1372.2530 |
| 13 | 986.9820 | 1216.6780 | 1384.2370 | 1383.4730 | 1889.3920 | 1104.9830 |
| 14 | 552.8920 | 821.6330 | 660.8320 | 564.9080 | 2062.2680 | 1172.2010 |
| 15 | 732.1130 | 1794.8280 | 278.4770 | 284.0450 | 23918.8590 | 3868.0200 |
| 16 | 1252.9350 | 1977.1500 | 480.4650 | 457.7360 | 4591.4380 | 1540.1610 |
| 17 | 1686.6390 | 1020.1720 | 266.3540 | 305.1670 | 8043.6240 | 998.3860 |
| 18 | 944.1890 | 823.2290 | 255.5600 | 307.8820 | 7980.8220 | 917.5620 |
| 19 | 673.4350 | 836.3990 | 422.6520 | 341.8280 | 4349.3850 | 8929.8380 |
| 20 | 718.5050 | 788.8700 | 659.8030 | 370.3450 | 5501.1340 | 2252.5270 |
